# Lentil (*Lens culinaris* Medik.) Flour Varieties as Promising New Ingredients for Gluten-Free Cookies

**DOI:** 10.3390/foods11142028

**Published:** 2022-07-08

**Authors:** Lívia Hajas, László Sipos, Éva Csajbókné Csobod, Márta Veresné Bálint, Réka Juhász, Csilla Benedek

**Affiliations:** 1 Department of Dietetics and Nutrition Science, Faculty of Health Science, Semmelweis University, 1088 Budapest, Hungary; hajas.livia@se-etk.hu (L.H.); csajbokne@se-etk.hu (É.C.C.); veresne@se-etk.hu (M.V.B.); benedek.csilla@se-etk.hu (C.B.); 2 Research Group of Cereal Science and Food Quality, Department of Applied Biotechnology and Food Science, Budapest University of Technology and Economics, 1111 Budapest, Hungary; 3 Department of Postharvest, Supply Chain, Commerce and Sensory Science, Institute of Food Science and Technology, Hungarian University of Agriculture and Life Sciences, 1118 Budapest, Hungary; sipos.laszlo@uni-mate.hu; 4 Centre for Economic and Regional Studies, Loránd Eötvös Research Network, Institute of Economics, 1111 Budapest, Hungary

**Keywords:** pulses, cookies, protein, antioxidant, texture, sensory properties

## Abstract

Monotony in a gluten-free (GF) diet can be a challenge because of a limited choice of acceptable cereal sources. This study investigates the potential of five types of differently coloured lentils in the development of GF cookies using rice flour as a reference. Raw materials (lentil flours) and cookies were characterised in terms of physicochemical parameters (e.g., crude protein content, total phenolics and flavonoids, antioxidant properties, colour, pH); additionally, geometry, baking loss and texture profile were determined for the cookies. A sensory acceptance test was also conducted to find out consumer preferences regarding rice versus different lentil cookies. Results showed that lentil cookies were superior to rice control in terms of higher crude protein (12.1–14.8 vs. 3.8 g/100 g), phenolic (136.5–342.3 vs. 61.5 mg gallic acid equivalents/100 g) and flavonoid (23.8–75.9 vs. 13.1 mg catechin equivalents/100 g) content and antioxidant capacity (0.60–1.81 vs. 0.35 mmol trolox equivalents/100 g), as well as lower hydroxymethyl-furfural content (<1 vs. 26.2 mg/kg). Consumers preferred lentil cookies to rice ones (overall liking: 6.1–7.0 vs. 5.6, significant differences for red and brown lentils), liking especially their taste (6.3–7.0 vs. 5.5). Depending on the target parameter, whether physicochemical or sensory, these lentil flours can be promising raw materials for GF bakery products.

## 1. Introduction

Celiac disease is an immune-mediated systemic disorder affecting approximately 1% of the world population (currently nearly 79.5 million people) [1]. Additionally, gluten consumption has been linked to the so-called ’gluten-related disorders’, including non-celiac gluten sensitivity, dermatitis herpetiformis, gluten ataxia and wheat allergy [1,2]. To prevent life-threatening conditions, strict adherence to a gluten-free (GF) diet is the primary choice of treatment.

Gluten-free diets often become nutritionally imbalanced. Because of the exclusion of gluten proteins, GF products typically have a lower protein content than conventional wheat flour-based versions, which should be covered by other sources. High intakes of sugar and saturated fatty acids are typical in GF diets. Deficiencies in several nutrients such as fibre, iron, zinc, magnesium, calcium, vitamin D, E and some of group B vitamins (B12 and B9) have also been reported [1,3,4,5,6]. This unfavourable dietary profile has been attributed to the nutritional composition of the GF products consumed and to imbalanced dietary habits [6].

It is therefore clear that the development of GF foods with enhanced nutritional value is an important and demanding task. The use of various alternative components and technologies may be an appropriate solution in this regard [2]. While new products emerge on the market regularly, many affected people find their diet rather difficult to follow. This is partly due to the limited accessibility and affordability of these products, especially those claimed to be ‘enriched’ or ‘functional’. According to recent reports, the main difficulties are encountered during social events and eating out [1,2,7,8].

Finding acceptable GF alternatives for celiacs in terms of both nutritive value and sensory acceptance is thus a continuous challenge. Among these products, biscuits and crackers represent a specific target segment, as their consumption was shown to be significantly higher among celiacs, often replacing bread [8]. It appears that affected people tend to consider biscuits and crackers as their main carbohydrate sources. Development of such products, possessing higher nutritional value than the traditional rice- or corn-based varieties, would be a good alternative in improving the dietary profile of celiac patients. Moreover, GF biscuits are easy-to-carry, ready-to-eat and on-the-go comfort foods that may support a strict diet. However, gluten is responsible for different important functional characteristics, thus mechanical and sensorial challenges have to be solved during the development of such products. While the absence of gluten proteins often makes development of GF bakery goods a very difficult task, it has a much lower impact on decrease of quality and in consumers’ acceptance in the case of cookies [1].

Nutrient intake of GF diet can be balanced by using pulses as raw materials in GF food production. The amino acid composition of legumes, such as lentils, can complement those of cereals (especially in the case of lysine and arginine), and they are also rich in bioactive compounds such as fibres and phytochemicals, including phenolic compounds [9,10,11].

Lentils (*Lens culinaris* Medik.) are known to come in various colours, including yellow, orange, red, green, brown or black, depending on the cultivar, the composition of the seed coats and cotyledons. The major commercial market classes of lentils are red (based on cotyledon colour of dehulled seeds) and green (based on seed coat colour) [9].

Compared to other pulses, lentils are characterised by higher protein, fibre and iron content, and have a remarkably high vitamin B content, especially folate (B9), which was reported to exceed that of rice by two magnitudes [12,13,14]. Their considerable carbohydrate content is composed partly by prebiotics; moreover, their resistant starch content may decrease the glycaemic index of foods [15,16,17]. As in most pulses, other bioactives like saponins, phytates, tannins and trypsin inhibitors are also present in lentils [18]. Lentils are also known to possess chymotrypsin-inhibitory activities [19]. Upon excessive consumption, their tannins and phytate may be detrimental for absorption of some minerals, especially iron and zinc [20]. However, these were shown to be inactivated or decreased by application of different cooking or baking technologies [21].

Compared to other pulses, lentils excel by their high phenolics content. The seed coats of lentils have a higher amount of flavan-3-ols, proanthocyanidins and some flavonols. Apart from polymeric phenolics (mostly procyanidins and prodelphinids), lentils contain both flavonoids (flavonols, flavones, flavanones, flavan-3-ols and anthocyanidins) and phenolic acids (hydroxybenzoic and hydroxycinnamic acids, stilbenes) [22,23]. Antioxidant capacity is highly determined by the colour of the lentils. In a study comparing brown, red, dark green, French green, black Beluga, dehulled and split red and yellow lentils, the highest in vitro polyphenol and flavonoid contents, as well as antioxidant capacities were measured for the black, dark green and French green types, while dehulled and split red and yellow lentils had the lowest performances. Cooked lentils had lower contents as well, while germination had a beneficial effect on these parameters [24]. Application of different cooking methods also resulted in the decrease of both free and bound phenolic acids [25].

According to recent scientific reports, it is possible to formulate acceptable GF biscuits using legumes, which may also enhance the nutritional quality, the antioxidant activity and the glycemic index [1]. However, despite their favourable properties, lentils are still underutilised in the area of GF biscuit-type products. A novel study investigates the utilisation of brown lentil flour in combination with wheat flour in concentrations of up to 25% lentil flour. The obtained cookies had better hardness and colour characteristics compared to the wheat product [26].

The aim of the present research is to develop and characterise lentil-based cookies that could be offered to celiac patients as nutritionally favourable GF snack alternatives. Five types of lentils of different colours (black, brown, green, red and yellow) were used as flour ingredients in cookies. The products were compared to a rice-containing reference (as rice is one of the most commonly used carbohydrate sources in GF diets).

## 2. Materials and Methods

### 2.1. Materials

Five lentil (*L. culinaris*) varieties—black (bl), brown (br), green (gr), red (re) and yellow (ye) (based on coat colours)—were used in this study. Rice flour (RF) (type: Nagykun white rice flour, Nagykun 2000 Mezőgazdasági Zrt.) was used as control. Lentils were purchased in a local market in Budapest, Hungary. The country of origin is Canada for black, brown, green and red lentils, and Turkey for dehulled and split yellow lentil.

Lentil flours were prepared using a Grindomix GM 200 knife mill (Retsch GmbH, Haan, Germany). A 250 g amount of lentil was ground using the ’hit’ function at 4000 rpm for 10 s then the ’cut’ function at 7500 rpm for 10 s. Whole grain lentil flour was then sieved using a 500 micrometre manual sieve and thus separated to lentil bran (LB) and lentil flour (LF).

Cookies were prepared based on the AACC Approved Method 10-50D [27]. Dough was prepared by one-stage mixing, in which margarine and flour were blended first for five minutes. Then the other components were added and mixing continued for 10 min. Dough was laminated (6 mm thickness), then cut with a circular cookie cutter (inner diameter 50 mm) and baked in an electrically heated oven for 10 min at 200 °C. Using the following portions and recipe in Table 1, ten cookies per type were prepared. The lentil product types (flours, brans and cookies) are listed with their abbreviations in Table 2. Their photos can be seen in Figures S1 and S2.

### 2.2. Methods

Colour, moisture, crude protein content, total polyphenol content, total flavonoid content and antioxidant capacity were determined in the case of lentil flours and cookies. Methods that were the same in flours and cookies therefore are summarised in Section 2.2.1. Determination of pH and hydroxymethyl-furfural content, texture profile analysis and sensorial evaluation were performed in the case of cookies and are introduced in Section 2.2.2. In the case of doughs, only texture profile analysis was performed.

#### 2.2.1. Methods Used in Both Cookie and Flour Samples

##### Colour Measurement

Instrumental colour measurement was performed with the Chroma Meter CR-410 (Konica Minolta, Inc., Tokyo, Japan). Five replicates were performed for each whole seed, flour and bran. Surface colour was measured for each individual cookie. Ten replicates (baked cookies) were obtained per sample type. The instrument was standardised against a reference white tile (CR-A44) before sample measurements. Results were expressed in the CIELab tristimuli coordinate system. L* is a measure of the lightness from black (0) to white (100), while a* describes the red–green colour range (a* > 0 indicates redness, a* < 0 indicates greenness), and b* describes the yellow–blue colour range (b* > 0 indicates yellowness, b* < 0 indicates blueness). To determine the total colour difference (∆E*_a,b_) between two samples, the following formula was used:

(1) $$∆E_{a,b}^{*}=\sqrt{{\left(L_{2}-L_{1}\right)}^{2}+{\left(a_{2}-a_{1}\right)}^{2}+{\left(b_{2}-b_{1}\right)}^{2}}$$

If ∆E*_a,b_ is less than 1, no colour difference is perceived to the human eye, while colour difference is clearly visible if ∆E*_a,b_ > 3 [28].

##### Determination of Moisture and Crude Protein Content

Prior to measurements, cookies were ground using a Grindomix GM 200 knife mill (Retsch GmbH, Haan, Germany): ’cut’ function was applied at 7500 rpm for 5 s, then ’hit’ function was applied at 4000 rpm for 5 s. The moisture content of samples was determined in triplicates with the air-oven drying method at 105 °C for 2 h. It was calculated based on weight differences before and after drying.

Nitrogen content of flours and ground cookies was determined with the Kjeldahl method in three parallel measurements using a Labtec^TM^ DT208 Digestor (FOSS Analytical Co., Ltd., Suzhou, China) unit and Kjeltec^TM^ 8200 equipment (FOSS Analytical Co., Ltd., Suzhou, China). Samples of 0.5 to 1 g were weighed and then digested in the presence of concentrated sulfuric acid and Se-based catalyst. A solution of boric acid was used to collect the distilled ammonia. Titrations were performed with standardised 0.1N hydrochloric acid. Protein conversion factor was 6.25 for lentil flours and cookies, and 5.95 for rice flour and cookies [29].

##### Total Polyphenol, Total Flavonoid Content and Antioxidant Capacity

Stock solutions for analytical measurements were prepared by extracting 2 g of homogenised ground material with 10 mL methanol:water:acetic acid (75:24.9:0.1) in a centrifuge tube. Extraction was performed by vortexing the tube for 1 min at 1800 rpm (relative centrifugal force: 7.3 g), then phases were separated by centrifugation at 4000 rpm (relative centrifugal force: 2468 g) for 5 min. Supernatants were pipetted in a normal flask, then the whole procedure was repeated on the residue and supernatants were merged. Five replicates were prepared for each analytical measurement.

Total polyphenol content (TPC) was determined with the Folin–Ciocalteu reagent [30], using gallic acid as standard. The TPC value was expressed as mg of gallic acid equivalents (GAE) per 100 g of cookie. The quantification of total flavonoid content (TFC) was carried out by means of the aluminium chloride colorimetric method [31], using catechin as a standard. The TFC was expressed as mg catechin equivalents (CE) per 100 g of cookie. The cupric(II) ion-reducing capacity (CUPRAC) method [32] was performed against trolox as standard. The results were expressed as mmol trolox equivalents (TE) per 100 g cookie. During each analytical measurement, the resulting colour was determined spectrophotometrically on a Helios Alpha spectrophotometer (Thermo Spectronic, Cambridge, England), using proper dilutions (twofold for TPC and CUPRAC) of the sample extracts.

#### 2.2.2. Methods Used in Cookie Samples

##### Baking Loss, Geometry

Ten cookies per sample type were weighed before baking (w_1_), and after cooling to room temperature (w_2_). Baking loss (BL) was calculated using the following equation:

(2) $$\mathrm{BL}=\frac{w_{1}-w_{2}}{w_{1}}\times 100$$

Dimensions of cookies were also measured using a measuring scale. Diameter (in mm) was obtained from end to end at the centre point of the cookies. By rotating them through 90°, two parallel readings were recorded. Cookies were cut into halves, then the height (in mm) was determined from the base to the upper surface at the centre point and the edges.

##### Texture Analysis

Texture of doughs and cookies was characterised using a Brookfield CT3 Texture Analyzer (Ametek Brookfield, Middleborough, MA, USA). In the case of doughs, it was equipped with the probe type TA44 (stainless steel cylinder, diameter: 4 mm). The test parameters were: total cycles 2, test speed 1 mm/s, target value 4 mm (distance reached by the cylindrical probe in the sample), trigger load 1 g. For cookies, texture profile analysis was performed with a TA9 probe (stainless steel needle, diameter: 1.5 mm). The test parameters were: total cycles 2, test speed 1 mm/s, target value 5 mm (distance reached by the needle probe in the sample), trigger load 4 g. Four samples were selected and the measurement was conducted in four replicates in the central position of each piece. Data recording and analysis of the texture profile were performed by using TexturePro CT v1.9 build 35 software (Ametek Brookfield, Middleborough, MA, USA). The units for certain texture parameters are given in the default form provided by the software. Based on the texture profile (load (g) in function of time (s)), the following parameters were determined: hardness (g) (maximum deformation force during the first mastication cycle) or hardness work (mJ) (area under peak, representative of energy required to obtain given deformation to target value); adhesive force (g) (force required to pull the needle probe away from the sample); cohesiveness (-) (the ratio of positive force during the second to that during the first compression); springiness (mm) (height that the food recovers during the time that elapses between the end of the first bite and the start of the second bite); gumminess (g) (calculated parameter: product of hardness × cohesiveness); chewiness (mJ) (calculated parameter: product of gumminess × springiness (equivalent to hardness × cohesiveness × springiness).

Determination of pH and Hydroxymethyl-Furfural Content

Five grams of ground cookie was dissolved in 10 mL distilled water. After 5 min, the concentration of hydrogen ions (pH value) was determined in triplicates using a Testo 206-pH2 digital pH meter (Testo SE & Co. KGaA, Lenzkirch, Germany). The hydroxymethyl-furfural content (HMF) value was measured based on the spectrophotometric method of Winkler. Two solutions were prepared for each cookie type and three replicates were performed for each solution (six replicates per cookie type). Cookie solutions were treated with a clarifying agent (Carrez solutions). Solutions of p-toluidine and barbituric acid were added, then the resultant colour was measured at 550 nm [33].

##### Sensory Tests

Sensory acceptance tests were conducted at a sensory laboratory (Hungarian University of Agriculture and Life Sciences, Institute of Food Science and Technology, Department of Postharvest, Supply Chain, Commerce and Sensory Science), which meets standard requirements [34,35]. The tests were designed, implemented and evaluated according to the international standard for consumer preference tests [36,37]. Products were coded with random numbers starting with non-zero. The samples were rotated to ensure balanced sample positioning. For testing the products, neutral mineral water was provided for all evaluations. Sensory test was performed by 61 students and workers (age ranging from 18 to 43 years old) recruited at Semmelweis University through internal advertisements. During the test, participants were asked to evaluate six different cookies based on their preference for a particular sensory attribute (colour, odour, hardness, crumbliness, taste, sweet taste, margarine taste, overall liking). On the score sheet, participants scored the cookies using a nine-category monotonic ascending hedonic response scale, with descriptive terms (1 = disliked very much, 9 = liked very much) and emoticon for each category [38]. A separate question was included to find out how likely the testing participants were to buy the cookie (1 = definitely no, 2 = probably no, 3 = maybe yes, maybe no, 4 = probably yes, 5 = definitely yes).

### 2.3. Data Analysis

In the case of texture parameters, data interquartile range was used as screening method prior to further analysis. Sixteen measured data were arranged in increasing order and the lowest and the highest two data were skipped during analysis. One-way ANOVA with significance level (α) of 0.05 were used to evaluate the effect of different cookie recipes on certain chemical, physicochemical, textural and sensory properties. Pairwise comparison was performed by Tukey HSD post-hoc test. Correlation among certain parameters (i.e., colour, antioxidants, etc.) was evaluated by calculation of Pearson correlation coefficient (r). Statistical analysis of measured data was performed using Statistica ver.13.5.0.17. (TIBCO Software Inc., Palo Alto, CA, USA) software.

## 3. Results and Discussion

### 3.1. Results Obtained for Raw Materials

The measurements performed on raw materials (whole grains and fractions resulting after sieving the differently coloured lentils) aimed at comparative evaluation of different lentils and different fractions, using rice flour (RF) as reference.

#### 3.1.1. Colour (L*, a*, b*) of Raw Materials

While lentil grains were all different (with one exception: a* for black/brown lentils) in terms of all colour parameters, these differences were partly diminished for a* of flours (fractions under 500 μm) and decreased even more for lentil brans (fractions above 500 μm), especially in the case of L* and b* (Table 3). This shows that the colour of the final product may strongly depend not only on the lentil variation, but also on the fraction used. Similar a* and b*, but higher L* values were reported for green lentil flours by a Turkish group; however, they used a 212 μm sieve for flour preparation [39].

Lentil samples showed huge visible colour difference compared to rice control as shown by the calculated total colour differences: ∆E*_a,b_ varied between 48.2 and 68.3, 36.9 and 46.1, and 44.0 and 55.0 in the case of seeds, flours and brans, respectively. When lentil varieties were compared to each other, total colour differences of whole seeds were lower, but still in a clearly visible range: (∆E*_a,b_ varied between 9.2 and 38.5). While all the brans showed similar brownish colour (∆E*_a,b_ between 0.8 and 13.9), the flours of different lentil varieties were easy to distinguish (∆E*_a,b_ between 3.1 and 16.7).

#### 3.1.2. Flour Yield

The yield of flour resulting upon grinding under controlled conditions and sieving on a 500 μm sieve may be an important factor in selection of the appropriate raw material for product development. The measurements (performed in two replicates) showed green lentil to produce the highest yield (56.6 ± 2.1%), while yellow lentil resulted in the lowest amount of flour (48.3 ± 0.4%). The yield of red, brown and black lentil flour was 50.7 ± 1.0%, 51.4 ± 3.4% and 54.7 ± 4.8%, respectively. However, there was no significant difference between the yield of different types of lentils (*p* > 0.05).

#### 3.1.3. Physicochemical Properties of Raw Materials

Moisture content results varied between 8.2% and 10.0% and showed significantly (*p* < 0.05) higher values for darker lentil flours: the lowest value being obtained for yellow lentil flour. Moisture contents somewhat higher than ours, between 10% and 14% were reported by Romano and co-workers [40]. Bubelová and co-workers [24] found moisture contents similar to ours, with the lowest moisture content for raw black Beluga lentils and the highest value for the raw French green variety. On the other hand, crude protein content was the highest for black, red and yellow lentil flours, while brown and green lentil flours performed worse. A Turkish study [39] reported 24.6% crude protein content for green lentil flour; Turfani et al. [10] reported 25.2% crude protein for the same product, which is in line with our findings. Fujiwara and co-workers [41] reported somewhat higher crude protein contents for split green and red lentil flours (28.1 and 28.9%, respectively). Others reported only small differences between green and red lentils for protein content [9]. Nevertheless, each of the lentil flours had a crude protein content 3–4 times higher if compared to rice control.

With regards to the presence of polyphenols, green lentils showed the highest results in terms of both total polyphenol and flavonoid content, as well as in vitro antioxidant properties (Table 4). Green lentils were superior to black lentils, even though the latter was expected to produce high values due to their colour as a possible indication of high polyphenol, i.e., anthocyanin content. Zou and co-workers [42] showed that antioxidant activities are highly related to colours, i.e., lentils with darker colour and higher phenolics contents showed higher contents in flavonoids, anthocyanins and enhanced antioxidant properties.

However, black lentils showed only the second highest antioxidant potential, followed by brown lentils, while red and yellow lentils were on the lowest end. Even these latter were superior to the rice control in terms of all antioxidant parameters. Bubelová and co-workers [24] investigated antioxidant properties of seven different types of raw lentils, finding Beluga black, followed by dark green and French green varieties to have the highest polyphenol and flavonoid content, as well as the highest antioxidant potential (DPPH (2,2-diphenyl-1-picrylhydrazyl) assay), while dehulled red and yellow lentils had the lowest performance.

It should be mentioned that the value reported in the literature for polyphenol content of green lentil flour is much lower (149.5 mg GAE/100 g) than our reported results [39]. In line with our results, French green lentils were also found to show the best performance in terms of total flavonoids when compared to red and green lentils, but total phenolic acid amounts were higher in green lentils, followed by French green and red lentils. DPPH antioxidant activity was, however, the highest for French green and green lentils, while red lentils had lower DPPH radical scavenging activity [43].

### 3.2. Results Obtained for Cookies

#### 3.2.1. Baking Loss, Geometry of the Cookies

Baking losses were not significantly different either within lentil cookies or compared to the rice reference (Table 5).

The geometry parameters of the cookies showed that red and yellow lentils yielded significantly flatter cookies, while there were no major differences between the rest of the lentil cookies. However, when compared to the rice reference product, practically all the lentil cookies were more applanate, which is generally undesired. Portman and co-workers [26] observed a similar trend for cookies prepared from wheat–lentil blends, which resulted in flatter and wider cookies compared to cookies made from 100% wheat flour. In contrast, Turkish researchers [39] found that wheat cookies were flatter than green lentil ones. Turfani et al. [10] observed reduced loaf volume when they increased the legume flour ratio to 10–12% or to 24% in wheat flour blends.

Baking loss during cookie processing is influenced by the water binding capacity of flour, which depends on both the starch and the protein content, structure and composition. Starch content, amylose to amylopectin ratio, size and structure of starch granules may affect water-binding capacity. The proteins that are able to form an elastic network during dough preparation increase the gas-holding capacity of dough, thus preventing volume loss during baking. Results indicate that, compared to rice, lentil flours possessed similar water-binding capacity but lower ability to form an elastic dough.

#### 3.2.2. Colour (L*, a*, b*) of the Cookies

Colour is an important factor in consumer choice. For biscuit-like products, brownish-reddish and yellowish colours are the desirable ones. As expected, dark-coloured lentils yielded significantly darker products compared to both red and yellow lentils and rice (Table 6). Red and yellow lentil cookies also differed significantly from each other and all the lentil products were significantly darker than rice cookies. The results were somewhat unexpected for lightly coloured yellow lentils, but are in line with the observations of other authors obtaining darker cookies with lentil–wheat blends compared to pure wheat products [26].

Red lentil cookies were also significantly more reddish than the other ones, black lentil cookies being on the other end of the range, but still reddish, while rice cookies showed intermediate reddish values. As regards yellowness, yellow lentil cookies reached the highest values, followed by red lentil and rice cookies. Black lentils produced products that were significantly less yellowish than all the rest. Investigating cookies made of green lentil flour, Oskaybaş-Emlek and co-workers [39] obtained cookies that were lighter (L* = 66.45) and more yellowish (b* = 34.11), but similar in reddish colour (a* = 4.46). Similarly to our results, Polat et al. [44] observed higher L* values for control crackers than for the ones prepared with germinated green lentil flour, L* decreasing with the increase of lentil content.

Colour differences are well reflected in ∆E*_a,b_ values which show perceivable differences for all the products, whether lentil- or rice-based. Black lentils resulted in cookies that have the most different colour compared to rice (46.4), while yellow lentils produced the lowest (19.1), but still a high colour difference. When lentil cookies are compared to each other, two pairs can be distinguished that have relatively lower colour differences, i.e., black-green (8.2) and yellow-red (8.0) lentil cookies.

Colour of celiac-friendly products proved to be an important factor in consumer preference and can be screened by computer vision. Rezagholi and co-worker [45] found that product colour was darker and their acceptance seriously decreased by replacing rice flour with purslane flour or quince seed gum above 50% and 1%, respectively.

#### 3.2.3. Physicochemical Properties of the Cookies

As regards chemical composition, rice cookies were shown to retain significantly more moisture than lentil ones. Black lentil cookies contained significantly less water than the rest, while moisture content of yellow lentil cookies was higher compared to the rest of the lentils (Table 7). Oskaybaş-Emlek and co-workers [39] also obtained higher moisture content in green lentil cookies than in wheat ones. Interestingly, the original moisture content of the lentil flours was not reflected in the baked products, suggesting that water retention due to the specificities of the dough structure has a greater impact on final moisture content than initial water content of the flours. As expected, crude protein results were the lowest for the rice products. As shown by others as well, the presence of lentils results in a significant increase in protein content even in different blends with cereals [26]. With regards to lentil types, only slight, but still significant differences were observed between lentil cookies, where black, red and yellow lentil cookies had the highest protein content. These differences were already observed for lentil flours, suggesting that they may have been retained during the baking process.

Antioxidant properties of baked products can be affected by multiple factors, including raw materials, Maillard reaction and caramelisation by-products as the most important ones. In the case of lentils, polyphenol content is the major factor determining antioxidant properties, which was also proven for lentil cookies. As in the case of flours, rice cookies had similarly low performance in terms of all the parameters investigated. Based on its specific colour, usually reflecting anthocyanin content, black lentil was expected to produce cookies with the strongest antioxidant properties; however, green lentils performed better for all the three indicators measured. Brown lentil cookies produced intermediary results, while red and yellow lentil products reached the lowest values, but still approximately double of those measured for rice cookies. As seen from the comparison with lentil flour results (Table 4), the antioxidant properties of lentils were transferred to baked products, with green lentils and yellow lentils at the highest and lowest end of the range, respectively. Very strong correlation (r > 0.94, *p* < 0.05) was observed between flours and cookies in the case of each antioxidant parameter (CUPRAC, TPC, TFC). Polat and co-workers [44] found a total phenolics content of 3.33 mg GAE/g and 0.19 mg QE/g total flavonoid content in crackers containing 15% germinated green lentil flour.

Turkish researchers [39] obtained lower results for TPC of green lentil cookies (117.5 mg GAE/100 g), but also their lentil flour had lower performance than the one used in this present study. Previous research using wheat–lentil blends in cookies found a considerable contribution of lentil ratio in the increase of antioxidant activity (FRAP assay), detecting phenolic acids that were absent in cookies made from 100% wheat flour [26]. Turfani and co-workers [10] confirmed that lentil flour enriched bread with lysine-rich proteins, phenolic compounds and lignans, and in general increased its antioxidant power. They measured 293 mg/100 g d.w. for a bread containing 24% green lentil flour.

A remarkable advantage of the lentil cookies was their low hydroxymethyl-furfural content. While this value was 26.2 ± 3.2 mg/kg for the rice control, all the lentil cookies had hydroxymethyl-furfural (HMF) contents under 1 mg/kg (Table 8). Taking into account the carcinogenic potential of this substance resulting from the Maillard reaction during baking [46], this should be a clear asset of lentil-based bakery products. The results are somewhat surprising given that rice cookies showed a significantly higher pH (9.27 ± 0.02) than all the lentil cookies (from 7.20 ± 0.05 for green lentil cookies to 7.72 ± 0.02 for red lentil cookies). Lower pH values have been known to facilitate the formation of hydroxymethyl-furfural [47]. Polat et al. [44] found similarly low HMF content for crackers enriched with germinated green lentil extract; however, their values were slightly higher (4.00 mg/100 g at 15% green lentil flour content). However, the HMF content of cookies can be affected by several parameters such as water activity, sugar composition, type and amount of free amino acids [48], antioxidant content or type of leavening agent [49]. Our results suggest that the high amount of antioxidants present in lentil flours can prevent formation of HMF during baking the cookies.

### 3.3. Textural Properties of Doughs and Cookies

The texture parameters calculated from the texture profile recorded in the case of doughs and baked cookies are summarised in Table 9 (dough) and Table 10 (cookie).

The lentil flour doughs were easy to work with, as their cohesiveness was higher and hardness work was lower than that of rice dough. However, stickiness (indicated by adhesive force) of lentil doughs was higher than that of rice dough (3.7 ± 0.9 g), especially in the case of the yellow (8.1 ± 0.8 g) and the green lentils (9.1 ± 1.3 g).

As seen from texture analysis results (Table 10), rice cookies were significantly softer (lower hardness) and less sticky (low adhesive force), crumblier (lower cohesiveness) and less springy (lower springiness) than any of the lentil cookies (except for black lentils in the case of hardness; however, here high standard deviations were obtained). Heterogeneity of the cookie structure resulted in uneven increase of load value during the first mastication cycle and caused great standard deviation in texture parameters. Lower hardness can be attributed to the higher moisture content of rice cookies. Green lentil cookies were also reported to have higher hardness values than wheat cookies [39]. With regards to gumminess, rice cookies did not differ significantly from black lentil cookies. Values obtained for chewiness showed that only red and yellow lentil cookies had significantly higher values compared to rice cookies.

Our results confirm previous observations that the use of lentils for cookie preparation increases the hardness of the product. Polat and co-workers [44] reported that crackers produced with 5 to 15% germinated green lentil flour showed higher hardness and lower acceptance among consumers. Portman et al. [26] observed that using lentil flour in addition to wheat flour cookies made the product harder, thinner and darker. Increased hardness of cookies can be explained by several factors, such as differences in protein and starch content and structure resulting in changes of water-binding capacity of flours used in cookie preparation.

Differences in hardness, however, appeared not to be detrimental in ranking rice and lentil cookies during sensory analysis (Table 11). The lower cohesiveness of rice products is reflected in the sensory test in lower crumbliness ranking values for rice cookies, but these differences are not statistically significant.

As regards differences between lentil types, yellow and red ones were harder than the other ones (except for black lentils), which apparently did not affect their sensory appreciation. Black lentils are also different from the rest in terms of adhesive force, and are different from red and yellow lentil cookies in terms of gumminess and chewiness, reaching lower values for all these parameters. However, for the majority of the texture parameters, there were no significant differences between black, brown and green lentils, producing generally lower values for the indicators measured. Nevertheless, these differences between red and yellow versus black, brown and green lentils were not reflected in sensory ranking test results for hardness and crumbliness, thus higher gumminess, chewiness, hardness or adhesive force seem not to be a perceivable disadvantage for red and yellow lentil cookies.

### 3.4. Sensory Properties of Cookies

A sensory acceptance test (Table 11) covering seven properties and overall liking was conducted on cookies to estimate sensory differences between lentil cookies and between lentil cookies and rice control in order to outline consumer attitude towards these products and purchase intention.

As regards colour, rice and yellow lentil cookies obtained the highest ranking; however, these results were not statistically different from those obtained for red or even brown lentil-based cookies. The green and black lentil-based samples received significantly lower scores compared to other cookies. In line with our results, Turkish researchers [44] found that the 15% addition of germinated green lentil significantly affected the colour of the crackers. Although lentils are known for their specific odour, no statistically significant differences were detected in odour ranking of the cookies, whether lentil or rice. The same applies for margarine taste, which is usually not regarded as desirable for a bakery product.

According to Polat and co-workers [44], the 15% addition of germinated green lentil affected the taste and flavour of crackers due to the strong smell of lentil. In our study, the taste of all lentil-based cookies except for green lentil products received a better score compared to rice control. However, there was no significant difference between the taste of different types of lentils (*p* > 0.05). Han et al. [50] obtained similar results. They found no statistically significant differences in flavour between green and red lentil-based crackers.

Acceptability of sweetness of rice cookies was ranked as significantly inferior to all lentil cookies, among which no differences were detected. As far as texture is concerned, the hardness of rice cookies was not ranked as significantly different from that of lentil ones. Crumbliness of brown lentil cookies was significantly more preferred than that of the rice product; otherwise, lentil cookies did not differ among each other. When expressing their overall liking, participants ranked red and brown lentil cookies significantly higher than rice cookies, but no significant liking differences were detected among lentil cookies. This is partly also reflected in purchasing intention: rice cookies obtained again the lowest ranking, while brown lentil cookies obtained the significantly highest results. Fujiwara et al. [41] reported similar results. They found no statistically significant differences in palatability between controls and pulse variants. While black lentil cookies were ranked somewhat lower, no differences were measured between green, red and yellow lentil cookies.

## 4. Conclusions

The present study investigated the potential of five types of differently coloured lentils in the development of GF cookies, using rice flour product as a reference. The lentils used as raw materials were different in terms of the colour parameters measured (L*, a*, b*); however, these differences were reduced in the case of lentil flours.

Rice flour had lower crude protein content than any of the lentil flours. Black, red and yellow lentil flours had the highest protein contents. Rice also had a worse performance in terms of in vitro antioxidant capacity, total phenolics and flavonoids, for which green lentils were superior to all the others.

Cookies made of lentil flours were generally flatter than those made of rice flour, while baking loss was similar. All the lentil-based products were darker than rice cookies, with perceivable differences among all the products. The original moisture content of the lentil flours was not reflected in the baked products, but all were inferior to the rice ones. The presence of lentils significantly improved the crude protein content of the cookies, the highest values being reached for black, red and yellow lentil cookies. The phenolic and flavonoid contents and antioxidant properties of lentil and rice flours were transferred to the baked products, with green lentils remaining superior to the others and rice products being at the lowest end of the range. A specific benefit of the lentil cookies was their low hydroxymethyl-furfural content in comparison with the rice control.

Rice cookies were significantly softer than lentil ones and showed lower hardness, springiness, cohesiveness and chewiness than any type of lentil cookies. The sensory acceptance test showed that differences in hardness between rice and lentil cookies were not detrimental during consumer preferences. Brown and red lentil products were more accepted than any other lentil types or the rice product.

Based on the physicochemical and sensory examination of cookies prepared from five lentil types, it can be concluded that any of the lentils enhances crude protein content, phenolic and flavonoid content and antioxidant capacity compared to rice as reference GF raw material. However, significant differences were observed between differently coloured lentils regarding all the properties investigated. Employment of fuzzy logic and image processing could be promising tools in determination of the optimum formulation [45].

Results indicated that, depending on the target parameter, whether physicochemical (protein content or antioxidant property), morphological (geometry, texture) or sensory, any of these lentil flours can be a promising raw material for GF bakery products, having nutritional and sensorial quality superior to rice. Thus, lentils are expected to gain growing importance in the development of new GF products, including cookies. However, the type of lentil should be selected accordingly during product development.

## Figures and Tables

**Table 1:** Cookie recipe.

Ingredient	Amount
Lentil flour (or rice flour in the case of control)	100.00 g
Powdered sugar	57.80 g
Margarine (with 70% fat content)	28.40 g
Salt	0.93 g
Sodium bicarbonate	1.11 g
Distilled water	7.11 g
Glucose solution (5 g/100 mL)	14.60 g

**Table 2:** Abbreviations used for lentil types and products.

	Flour	Bran	Dough	Cookies
Black lentil	bl-LF	bl-LB	bl-LF-D	bl-LF-C
Brown lentil	br-LF	br-LB	br-LF-D	br-LF-C
Green lentil	gr-LF	gr-LB	gr-LF-D	gr-LF-C
Red lentil	re-LF	re-LB	re-LF-D	re-LF-C
Yellow lentil	ye-LF	ye-LB	ye-LF-D	ye-LF-C
Rice (control)	RF	–	RF-D	RF-C

**Table 3:** Colour parameters of whole grains, brans and flours from different lentil varieties compared to white rice flour.

Sample	L*	a*	b*
bl-WLS	25.26 ± 0.11 a	1.09 ± 0.03 b	−2.25 ± 0.04 a
br-WLS	39.70 ± 0.59 d	1.12 ± 0.17 b	11.37 ± 0.32 c
gr-WLS	31.10 ± 0.36 b	−0.03 ± 0.11 a	4.76 ± 0.22 b
re-WLS	38.13 ± 0.55 c	20.03 ± 0.19 d	28.68 ± 0.56 e
ye-WLS	46.89 ± 0.45 e	3.18 ± 0.17 c	26.82 ± 0.70 d
RF	92.27 ± 1.19 f	−0.60 ± −0.03 d	11.01 ± 0.11 b
bl-LF	46.19 ± 0,67 a	−1.56 ± 0.02 ab	10.23 ± 0.21 a
br-LF	53.22 ± 0.27 c	−1.49 ± 0.02 bc	15.48 ± 0.20 d
gr-LF	51.96 ± 0.22 b	−1.75 ± 0.13 a	12.62 ± 0.15 c
ye-LF	56.63 ± 0.31 e	−1.30 ± 0.05 c	20.69 ± 0.21 e
re-LF	54.54 ± 0.19 d	7.36 ± 0.22 e	21.65 ± 0.19 f
bl-LB	49.17 ± 0.83 b	2.16 ± 0.20 c	26.30 ± 0.51 a
br-LB	50.11 ± 0.32 b	1.14 ± 0.05 a	26.51 ± 0.31 a
gr-LB	50.13 ± 0.72 b	1.72 ± 0.31 bc	25.98 ± 0.32 a
re-LB	47.31 ± 0.70 a	14.69 ± 0.44 d	26.68 ± 0.62 a
ye-LB	51.69 ± 0.95 c	1.56 ± 0.21 ab	28.01 ± 0.60 b

RF: rice flour, LF: lentil flour, LB: lentil bran, WLS: whole lentil seed, bl: black, br: brown, gr: green, re: red, ye: yellow. Data presented as mean ± standard deviation (*n* = 5). Within each column and sample type (WLS, LF or LB) different letters represent the significant difference between means (*p* < 0.05; one-way ANOVA, Tukey’s HSD test).

**Table 4:** Moisture and crude protein (CP) content, cupric ion-reducing antioxidant capacity (CUPRAC), total polyphenol (TPC) and total flavonoid (TFC) content of lentil flours compared to white rice flour.

Sample	Moisture (g/100 g) *n* = 3	CP (g/100 g) *n* = 3	CUPRAC (mmol TE/100 g) *n* = 5	TPC (mg GAE/100 g) *n* = 5	TFC (mg CE/100 g) *n* = 5
RF	9.3 ± 0.1 b	6.5 ± 0.1 a	0.29 ± 0.02 a	44.0 ± 3.8 a	14.3 ± 1.9 a
bl-LF	9.9 ± 0.1 d	25.7 ± 0.1 c	1.09 ± 0.02 d	244.4 ± 7.1 e	34.2 ± 2.6 c
br-LF	9.9 ± 0.1 d	21.4 ± 0.3 b	0.77 ± 0.04 c	197.2 ± 5.7 d	39.0 ± 2.7 c
gr-LF	10.0 ± 0.1 d	22.5 ± 0.1 b	1.58 ± 0.04 e	337.3 ± 5.2 f	63.3 ± 2.7 d
re-LF	9.5 ± 0.0 c	24.8 ± 1.1 c	0.35 ± 0.01 ab	151.1 ± 2.6 c	24.6 ± 5.1 b
ye-LF	8.2 ± 0.1 a	24.8 ± 0.2 c	0.36 ± 0.03 b	113.2 ± 4.3 b	24.7 ± 3.2 b

GAE: gallic acid equivalent, TE: trolox equivalent, CE: catechin equivalent, RF: rice flour, LF: lentil flour, bl: black, br: brown, gr: green, re: red, ye: yellow. Data presented as mean ± standard deviation. Within each column, different letters represent the significant difference between means (*p* < 0.05; one-way ANOVA, Tukey’s HSD test).

**Table 5:** Baking loss, diameter and height of rice and lentil cookies.

Sample	Baking Loss, (%), *n* = 10	Diameter (mm), *n* = 20	Height (mm), *n* = 30	Diameter/ Height
RF-C	14.0 ± 0.8 a	59.9 ± 1.3 a	9.8 ± 1.0 d	6.1
bl-LF-C	13.3 ± 0.7 a	63.0 ± 2.9 b	9.1 ± 0.7 c	6.9
br-LF-C	13.7 ± 0.7 a	62.0 ± 1.9 ab	8.6 ± 0.7 c	7.2
gr-LF-C	14.1 ± 0.7 a	62.6 ± 2.1 b	9.0 ± 0.6 c	7.0
re-LF-C	14.2 ± 0.8 a	68.6 ± 2.8 c	7.3 ± 0.5 b	9.4
ye-LF-C	13.9 ± 0.8 a	68.9 ± 2.6 c	6.5 ± 0.6 a	10.6

RF-C: cookie made with rice flour, LF-C: cookie made with lentil flour, bl: black, br: brown, gr: green, re: red, ye: yellow. Data presented as mean ± standard deviation. Within each column, different letters represent the significant difference between means (*p* < 0.05; one-way ANOVA, Tukey’s HSD test).

**Table 6:** Colour parameters of lentil and rice cookies.

Sample	L*	a*	b*
RF-C	72.68 ± 1.98 f	5.23 ± 1.22 b	34.37 ± 1.22 d
bl-LF-C	33.79 ± 0.61 a	1.69 ± 0.36 a	9.31 ± 0.35 a
br-LF-C	45.20 ± 0.60 c	8.96 ± 0.65 c	24.28 ± 0.29 c
gr-LF-C	38.25 ± 0.55 b	4.62 ± 0.49 b	15.48 ± 0.23 b
re-LF-C	51.50 ± 1.08 d	18.24 ± 0.32 e	35.72 ± 0.91 d
ye-LF-C	55.12 ± 2.30 e	11.63 ± 1.60 d	38.30 ± 2.04 e

RF-C: cookie made with rice flour, LF-C: cookie made with lentil flour, bl: black, br: brown, gr: green, re: red, ye: yellow. Data presented as mean ± standard deviation (*n* = 10). Within each column, different letters represent the significant difference among means (*p* < 0.05; one-way ANOVA, Tukey’s HSD test).

**Table 7:** Moisture and crude protein (CP) content, cupric ion-reducing antioxidant capacity (CUPRAC), total polyphenol (TPC) and total flavonoid (TFC) content of lentil and rice cookies.

Sample	Moisture (g/100 g) *n* = 3	CP (g/100 g) *n* = 3	CUPRAC (mmol TE/100 g) *n* = 5	TPC (mg GAE/100 g) *n* = 5	TFC (mg CE/100 g) *n* = 5
RF-C	5.4 ± 0.0 d	3.8 ± 0.1 a	0.35 ± 0.04 a	61.5 ± 1.7 a	13.1 ± 2.9 a
bl-LF-C	4.3 ± 0.1 a	14.6 ± 0.1 de	1.57 ± 0.08 e	342.3 ± 13.3 e	56.9 ± 2.6 c
br-LF-C	4.5 ± 0.0 b	12.1 ± 0.2 b	1.29 ± 0.08 d	269.5 ± 4.8 d	56.7 ± 3.4 c
gr-LF-C	4.5 ± 0.0 b	13.2 ± 0.1 c	1.81 ± 0.09 f	399.9 ± 14.6 f	75.9 ± 6.9 d
re-LF-C	4.4 ± 0.0 b	14.8 ± 0.0 e	0.82 ± 0.02 c	196.3 ± 10.1 c	24.6 ± 1.4 b
ye-LF-C	4.9 ± 0.0 c	14.4 ± 0.2 d	0.60 ± 0.01 b	136.5 ± 3.5 b	23.8 ± 2.9 b

GAE: gallic acid equivalent, TE: trolox equivalent, CE: catechin equivalent, RF-C: cookie made with rice flour, LF-C: cookie made with lentil flour, bl: black, br: brown, gr: green, re: red, ye: yellow. Data presented as mean ± standard deviation. Within each column, different letters represent the significant difference between means (*p* < 0.05; one-way ANOVA, Tukey’s HSD test).

**Table 8:** pH and hydroxymethyl-furfural content of lentil and rice cookies.

	pH *n* = 3	HMF (mg/kg) *n* = 6
RF-C	9.27 ± 0.02 c	26.2 ± 3.2
bl-LF-C	7.26 ± 0.01 a	<1
br-LF-C	7.63 ± 0.08 b	<1
gr-LF-C	7.20 ± 0.05 a	<1
re-LF-C	7.72 ± 0.02 b	<1
ye-LF-C	7.64 ± 0.01 b	<1

RF-C: cookie made with rice flour, LF-C: cookie made with lentil flour, bl: black, br: brown, gr: green, re: red, ye: yellow. Data presented as mean ± standard deviation. Within each column, different letters represent the significant difference between means (*p* < 0.05; one-way ANOVA, Tukey’s HSD test).

**Table 9:** Textural properties of raw dough of rice control and lentil cookies.

	Hardness Work (mJ)	Adhesive Force (g)	Cohesiveness (-)	Springiness (mm)
RF-D	2.8 ± 0.4 d	3.7 ± 0.9 a	0.06 ± 0.01 a	5.9 ± 0.3 bc
bl-LF-D	1.5 ± 0.1 b	7.3 ± 0.9 bc	0.14 ± 0.01 c	5.3 ± 0.4 a
br-LF-D	1.5 ± 0.1 b	7.7 ± 0.7 c	0.14 ± 0.01 c	5.9 ± 0.4 c
gr-LF-D	2.5 ± 0.3 c	9.1 ± 1.3 d	0.12 ± 0.00 b	5.0 ± 0.5 a
re-LF-D	1.3 ± 0.1 b	6.3 ± 0.8 b	0.17 ± 0.01 d	5.4 ± 0.2 ab
ye-LF-D	0.8 ± 0.1 a	8.1 ± 0.8 cd	0.21 ± 0.02 e	5.3 ± 0.5 a

RF-D: dough made with rice flour, LF-D: dough made with lentil flour, bl: black, br: brown, gr: green, re: red, ye: yellow. Data presented as mean ± standard deviation (*n* = 12). Within each column, different letters represent the significant difference among means (*p* < 0.05; one-way ANOVA, Tukey’s HSD test).

**Table 10:** Textural properties of rice control and lentil cookies.

	Hardness (g)	Adhesive Force (g)	Cohesiveness (-)	Springiness (mm)	Gumminess (g)	Chewiness (mJ)
RF-C	73.0 ± 45.9 a	11.3 ± 6.2 a	0.09 ± 0.01 a	1.0 ± 0.3 a	7.3 ± 4.4 a	0.08 ± 0.06 a
bl-LF-C	143.9 ± 39.8 ab	38.4 ± 9.9 b	0.15 ± 0.02 bc	1.9 ± 0.7 ab	20.5 ± 5.2 ab	0.40 ± 0.20 a
br-LF-C	216.5 ± 99.2 b	43.9 ± 11.7 bc	0.16 ± 0.02 c	2.0 ± 0.7 ab	34.3 ± 16.4 b	0.77 ± 0.45 ab
gr-LF-C	240.5 ± 89.5 bc	53.1 ± 24.1 bc	0.12 ± 0.02 b	2.1 ± 0.8 b	29.7 ± 13.5 b	0.68 ± 0.61 a
re-LF-C	363.8 ± 164.2 cd	55.6 ± 23.2 bc	0.15 ± 0.01 bc	2.9 ± 1.3 b	55.6 ± 27.7 c	1.59 ± 1.17 c
ye-LF-C	469.4 ± 184.8 d	60.5 ± 17.3 c	0.15 ± 0.03 c	2.5 ± 1.2 b	66.3 ± 15.2 c	1.54 ± 0.80 bc

RF-C: cookie made with rice flour, LF-C: cookie made with lentil flour, bl: black, br: brown, gr: green, re: red, ye: yellow. Data presented as mean ± standard deviation (*n* = 12). Within each column, different letters represent the significant difference among means (*p* < 0.05; one-way ANOVA, Tukey’s HSD test).

**Table 11:** Sensory properties of rice control and lentil cookies. RF-C: cookie made with rice flour, LF-C: cookie made with lentil flour, bl: black, br: brown, gr: green, re: red, ye: yellow.

	RF-C	bl-LF-C	br-LF-C	gr-LF-C	re-LF-C	ye-LF-C
Colour	7.1 ± 1.4 b	5.1 ± 2.5 a	6.8 ± 1.6 b	5.5 ± 2.0 a	6.8 ± 1.9 b	7.3 ± 1.6 b
Odour	5.3 ± 1.7 a	5.0 ± 1.8 a	5.7 ± 1.8 a	5.1 ± 1.7 a	5.8 ± 1.7 a	5.9 ± 1.8 a
Hardness	6.3 ± 2.0 ab	5.8 ± 2.3 ab	6.3 ± 2.0 b	5.2 ± 2.3 a	6.0 ± 2.1 ab	5.5 ± 2.2 ab
Crumbliness	5.6 ± 2.4 a	6.1 ± 2.0 ab	6.8 ± 1.7 b	6.3 ± 2.0 ab	6.4 ± 1.9 ab	6.3 ± 2.2 ab
Taste	5.5 ± 2.4 a	6.5 ± 1.7 b	7.0 ± 1.7 b	6.3 ± 1.7 ab	7.0 ± 1.5 b	6.5 ± 2.0 b
Sweet taste	6.0 ± 2.0 a	6.4 ± 1.9 ab	6.8 ± 1.9 ab	6.3 ± 1.9 ab	7.0 ± 1.6 b	7.0 ± 1.7 b
Margarine taste	5.8 ± 1.8 a	5.8 ± 2.0 a	6.0 ± 2.0 a	5.7 ± 2.0 a	6.0 ± 1.7 a	5.7 ± 2.0 a
Overall liking	5.6 ± 2.3 a	6.5 ± 1.8 ab	7.0 ± 1.6 b	6.1 ± 1.7 ab	6.8 ± 1.6 b	6.2 ± 1.9 ab
Purchase intention	2.6 ± 1.3 a	2.8 ± 1.3 ab	3.6 ± 1.1 c	3.0 ± 1.1 abc	3.4 ± 1.1 cb	3.1 ± 1.2 abc

Data presented as mean ± standard deviation (*n* = 61). Within each row, different letters represent the significant difference among means (*p* < 0.05; one-way ANOVA, Tukey’s HSD test).

## Data Availability

The data presented in this study are available on request from the corresponding author.

## Supplementary Materials

The following supporting information can be downloaded at: https://www.mdpi.com/article/10.3390/foods11142028/s1, Figure S1: Whole seed, flour and bran of five types of differently coloured lentils; Figure S2: Doughs and cookies made of rice and differently coloured lentil types.

## References

[1] Di Cairano M., Galgano F., Tolve R., Caruso M.C., Condelli N. (2018). Focus on Gluten Free Biscuits: Ingredients and Issues. Trends Food Sci. Technol..

[2] Melini V., Melini F. (2019). Gluten-Free Diet: Gaps and Needs for a Healthier Diet. Nutrients.

[3] Jnawali P., Kumar V., Tanwar B. (2016). Celiac Disease: Overview and Considerations for Development of Gluten-Free Foods. Food Sci. Hum. Wellness.

[4] Naqash F., Gani A., Gani A., Masoodi F.A. (2017). Gluten-Free Baking: Combating the Challenges—A Review. Trends Food Sci. Technol..

[5] Vici G., Belli L., Biondi M., Polzonetti V. (2016). Gluten Free Diet and Nutrient Deficiencies: A Review. Clin. Nutr..

[6] Cardo A., Churruca I., Lasa A., Navarro V., Vázquez-Polo M., Perez-Junkera G., Larretxi I. (2021). Nutritional Imbalances in Adult Celiac Patients Following a Gluten-Free Diet. Nutrients.

[7] Szűcs V., Fazakas Z., Farr A.M., Tarcea M. (2019). Quality of Life of Consumers Following a Gluten-Free Diet. Results of a Questionnaire Survey in Hungary and Romania. Orv. Hetil..

[8] Valitutti F., Iorfida D., Anania C., Trovato C.M., Montuori M., Cucchiara S., Catassi C. (2017). Cereal Consumption among Subjects with Celiac Disease: A Snapshot for Nutritional Considerations. Nutrients.

[9] Khazaei H., Subedi M., Nickerson M., Martínez-Villaluenga C., Frias J., Vandenberg A. (2019). Seed Protein of Lentils: Current Status, Progress, and Food Applications. Foods.

[10] Turfani V., Narducci V., Durazzo A., Galli V., Carcea M. (2017). Technological, Nutritional and Functional Properties of Wheat Bread Enriched with Lentil or Carob Flours. LWT-Food Sci. Technol..

[11] Mitchell D.C., Marinangeli C.P.F., Pigat S., Bompola F., Campbell J., Pan Y., Curran J.M., Cai D.J., Jaconis S.Y., Rumney J. (2021). Pulse Intake Improves Nutrient Density among US Adult Consumers. Nutrients.

[12] U.S. Department of Agriculture Food Data Central. https://fdc.nal.usda.gov/.

[13] Sen Gupta D., Thavarajah D., Knutson P., Thavarajah P., McGee R.J., Coyne C.J., Kumar S. (2013). Lentils (*Lens culinaris* L.), a Rich Source of Folates. J. Agric. Food Chem..

[14] Hall C., Hillen C., Garden Robinson J. (2017). Composition, Nutritional Value, and Health Benefits of Pulses. Cereal Chem..

[15] Ferawati F., Hefni M., Witthöft C. (2019). Flours from Swedish Pulses: Effects of Treatment on Functional Properties and Nutrient Content. Food Sci. Nutr..

[16] Siva N., Thavarajah P., Thavarajah D. (2018). The Impact of Processing and Cooking on Prebiotic Carbohydrates in Lentil. J. Food Compos. Anal..

[17] Siva N., Johnson C.R., Richard V., Jesch E.D., Whiteside W., Abood A.A., Thavarajah P., Duckett S., Thavarajah D. (2018). Lentil (*Lens culinaris* Medikus) Diet Affects the Gut Microbiome and Obesity Markers in Rat. J. Agric. Food Chem..

[18] Patterson C.A., Curran J., Der T. (2017). Effect of Processing on Antinutrient Compounds in Pulses. Cereal Chem..

[19] Landi N., Pacifico S., Piccolella S., Di Giuseppe A.M.A., Mezzacapo M.C., Ragucci S., Iannuzzi F., Zarrelli A., Di Maro A. (2015). Valle Agricola Lentil, an Unknown Lentil (*Lens culinaris* Medik.) Seed from Southern Italy as a Novel Antioxidant and Prebiotic Source. Food Fuction.

[20] Margier M., Georgé S., Hafnaoui N., Remond D., Nowicki M., Du Chaffaut L., Amiot M.J., Reboul E. (2018). Nutritional Composition and Bioactive Content of Legumes: Characterization of Pulses Frequently Consumed in France and Effect of the Cooking Method. Nutrients.

[21] Foschia M., Horstmann S.W., Arendt E.K., Zannini E. (2017). Legumes as Functional Ingredients in Gluten-Free Bakery and Pasta Products. Annu. Rev. Food Sci. Technol..

[22] Ganesan K., Xu B. (2017). Polyphenol-Rich Lentils and Their Health Promoting Effects. Int. J. Mol. Sci..

[23] Paucean A., Moldovan O.P., Mureșan V., Socaci S.A., Dulf F.V., Alexa E., Man S.M., Mureșan A.E., Muste S. (2018). Folic Acid, Minerals, Amino-Acids, Fatty Acids and Volatile Compounds of Green and Red Lentils. Folic Acid Content Optimization in Wheat-Lentils Composite Flours. Chem. Cent. J..

[24] Bubelová Z., Sumczynski D., Salek R.N. (2018). Effect of Cooking and Germination on Antioxidant Activity, Total Polyphenols and Flavonoids, Fiber Content, and Digestibility of Lentils (*Lens culinaris* L.). J. Food Process. Preserv..

[25] Liu Y., Ragaee S., Marcone M.F., Abdel-Aal E.S.M. (2020). Composition of Phenolic Acids and Antioxidant Properties of Selected Pulses Cooked with Different Heating Conditions. Foods.

[26] Portman D., Maharjan P., McDonald L., Laskovska S., Walker C., Irvin H., Blanchard C., Naiker M., Panozzo J.F. (2020). Nutritional and Functional Properties of Cookies Made Using Down-Graded Lentil—A Candidate for Novel Food Production and Crop Utilization. Cereal Chem..

[27] AACC (American Association of Cereal Chemists) (2000). Approved Methods of the American Association of Cereal Chemists.

[28] CIE (Commission Internationale de l’Éclairage) (2004). Technical Report: Colorimetry.

[29] FAO (Food and Agriculture Organization of the United Nations) (2003). Food Energy—Methods of Analysis and Conversion Factors.

[30] Singleton V.L., Rossi J.A. (1965). Colorimetry of Total Phenolics with Phosphomolybdic-Phosphotungstic Acid Reagents. Am. J. Enol. Vitic..

[31] Chang C.-C., Yang M.-H., Wen H.-M., Chern J.-C. (2002). Estimation of Total Flavonoid Content in Propolis by Two Complementary Colorimetric Methods. J. Food Drug Anal..

[32] Apak R., Güçlü K., Özyürek M., Karademir S.E. (2004). Novel Total Antioxidant Capacity Index for Dietary Polyphenols and Vitamins C and E, Using Their Cupric Ion Reducing Capability in the Presence of Neocuproine: CUPRAC Method. J. Agric. Food Chem..

[33] Zappalà M., Fallico B., Arena E., Verzera A. (2005). Methods for the Determination of HMF in Honey: A Comparison. Food Control.

[34] (2007). Sensory Analysis—General Guidance for the Design of Test Rooms.

[35] (2014). Sensory Analysis—General Guidance for the Design of Test Rooms—Amendment 1.

[36] (2014). Sensory Analysis—Methodology—General Guidance for Conducting Hedonic Tests with Consumers in a Controlled Area.

[37] (2020). Sensory Analysis—Methodology—General Guidance for Conducting Hedonic Tests with Consumers in a Controlled Area—Amendment 1.

[38] (2003). Sensory Analysis—Guidelines for the Use of Quantitative Response Scales.

[39] Oskaybaş-Emlek B., Özbey A., Kahraman K. (2021). Effects of Germination on the Physicochemical and Nutritional Characteristics of Lentil and Its Utilization Potential in Cookie-Making. J. Food Meas. Charact..

[40] Romano A., Gallo V., Ferranti P., Masi P. (2021). Lentil Flour: Nutritional and Technological Properties, in Vitro Digestibility and Perspectives for Use in the Food Industry. Curr. Opin. Food Sci..

[41] Fujiwara N., Hall C., Jenkins A.L. (2017). Development of Low Glycemic Index (GI) Foods by Incorporating Pulse Ingredients into Cereal-Based Products: Use of in Vitro Screening and in Vivo Methodologies. Cereal Chem..

[42] Zou Y., Chang S.K.C., Gu Y., Qian S.Y. (2011). Antioxidant Activity and Phenolic Compositions of Lentil (*Lens culinaris* Var. Morton) Extract and Its Fractions. J. Agric. Food Chem..

[43] Lee S.-Y., Yeo Y.-S., Park S.-Y., Lee S.-G., Lee S.-M., Cho H.-S., Chung N.-J., Oh S.-W. (2017). Compositional Analysis of Lentil (*Lens culinaris*) Cultivars Related to Colors and Their Antioxidative Activity. Plant Breed. Biotechnol..

[44] Polat H., Dursun Capar T., Inanir C., Ekici L., Yalcin H. (2020). Formulation of Functional Crackers Enriched with Germinated Lentil Extract: A Response Surface Methodology Box-Behnken Design. LWT.

[45] Rezagholi F., Hesarinejad M.A. (2017). Integration of Fuzzy Logic and Computer Vision in Intelligent Quality Control of Celiac-Friendly Products. Procedia Comput. Sci..

[46] Shapla U.M., Solayman M., Alam N., Khalil M.I., Gan S.H. (2018). 5-Hydroxymethylfurfural (HMF) Levels in Honey and Other Food Products: Effects on Bees and Human Health. Chem. Cent. J..

[47] Delgado-Andrade C., Rufián-Henares J.A., Morales F.J. (2009). Hydroxymethylfurfural in Commercial Biscuits Marketed in Spain. J. Food Nutr. Res..

[48] Sharma P., Gujral H.S. (2014). Cookie Making Behavior of Wheat-Barley Flour Blends and Effects on Antioxidant Properties. LWT-Food Sci. Technol..

[49] Švecová B., Mach M. (2017). Content of 5-Hydroxymethyl-2-Furfural in Biscuits for Kids. Interdiscip. Toxicol..

[50] Han J., Janz J.A.M., Gerlat M. (2010). Development of Gluten-Free Cracker Snacks Using Pulse Flours and Fractions. Food Res. Int..

