# Supplementary figures and images for: Lentil (Lens culinaris Medik.) Flour Varieties as Promising New Ingredients for Gluten-Free Cookies
Source: Foods. 2022 Jul 8;11(14):2028. doi: 10.3390/foods11142028 (PMC9317262; doi:10.3390/foods11142028)

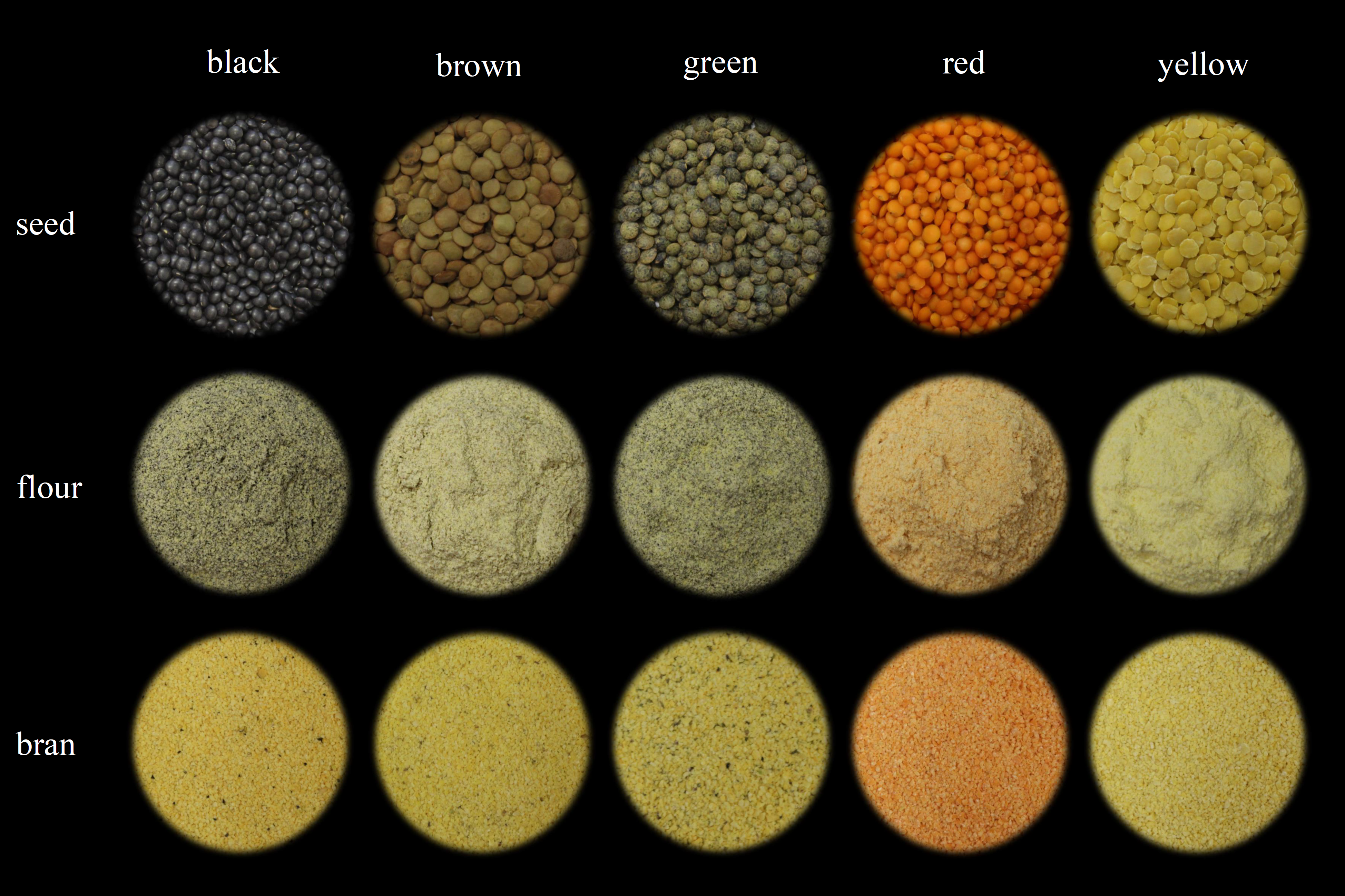

Supplement: Supplementary file 1 [file foods-11-02028-s001.zip › Supplement Foods_1794575/Figure S1.png]

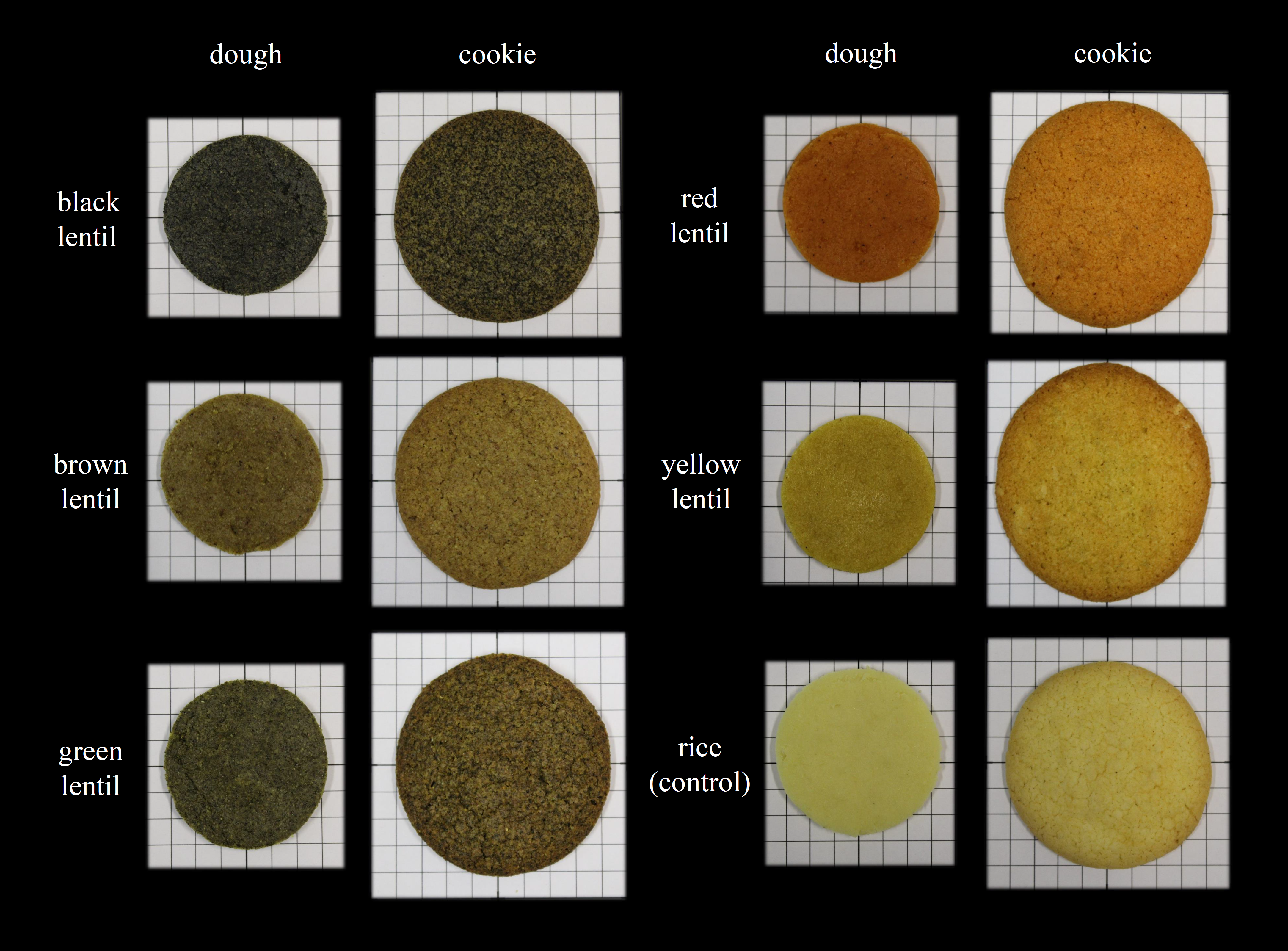

Supplement: Supplementary file 1 [file foods-11-02028-s001.zip › Supplement Foods_1794575/Figure S2.png]
